# Neuroprotective effects of ellorarxine in neuronal models of degeneration

**DOI:** 10.3389/fnins.2024.1422294

**Published:** 2024-09-10

**Authors:** Azita Kouchmeshky, Andrew Whiting, Peter McCaffery

**Affiliations:** ^1^School of Medicine, Medical Sciences and Nutrition, Institute of Medical Sciences, University of Aberdeen, Aberdeen, United Kingdom; ^2^Department of Chemistry, Science Laboratories, Durham University, Durham, United Kingdom

**Keywords:** retinoic acid, amyotrophic lateral sclerosis, excitotoxicity, proteostasis, stress granules

## Abstract

**Introduction:**

Retinoic acid (RA) was first recognised to be important for the central nervous system (CNS) in its developmental regulatory role and, given this action, it has been proposed in the adult CNS to regulate plasticity and promote regeneration. These types of roles have included support of neurogenesis, induction of neurite outgrowth, and protection from neuronal death. These functions are predominantly mediated by the retinoic acid receptor (RAR) transcription factor, and hence agonists for the RARs have been tested in a variety of models of neurodegeneration. This present study employs several *in vitro* models less explored for the action of RAR agonists to reverse neurodegeneration.

**Methods:**

A series of assays are used in which neuronal cells are placed under the types of stress that have been linked to neurodegeneration, in particular amyotrophic lateral sclerosis (ALS), and the neuroprotective influence of a new potent agonist for RAR, ellorarxine, is tested out. In these assays, neuronal cells were subjected to excitotoxic stress induced by glutamate, proteostasis disruption caused by epoxomicin, and oxidative stress leading to stress granule formation triggered by sodium arsenite.

**Results:**

Ellorarxine effectively reversed neuronal death in excitotoxic and proteostasis disruption assays and mitigated stress granule formation induced by sodium arsenite. This study also highlights for the first time the novel observation of RAR modulation of stress granules, although it is unknown whether this change in stress granules will be neuroprotective or potentially regenerative. Furthermore, the distribution of RAR agonists following intraperitoneal injection was assessed in mice, revealing preferential accumulation in the central nervous system, particularly in the spinal cord, compared to the liver. Gene expression studies in the spinal cord demonstrated that ellorarxine induces transcriptional changes at a low dose (0.01 mg/kg).

**Discussion:**

These findings underscore the therapeutic potential of RAR agonists, such as ellorarxine, for ALS and potentially other neurodegenerative diseases.

## Introduction

Amyotrophic lateral sclerosis (ALS) is a progressive and fatal motor neuron degenerative disease characterised by the selective loss of both upper and lower motor neurons, leading to progressive muscle weakness and eventual death. Most ALS cases are sporadic with unknown causes; however, a small number are linked to a heterogeneous set of gene mutations. The disease mechanisms include impairment in molecular trafficking, neuroinflammation, ER stress, excitotoxicity, protein aggregation and UPS dysfunction, mitochondrial dysfunction, and oxidative damage (Nagley et al., 2010). ALS likely involves interactions among these dysregulated mechanisms rather than a single cause.

Excitotoxicity, mediated by excessive glutamate release as the main excitatory neurotransmitter, leads to subsequent Ca^2+^ overload, excessive free radical generation, mitochondrial permeability transition activation, and neuronal death (Dong et al., 2009). Excitotoxicity is a significant contributor to ALS pathology, along with other neurodegenerative disorders including Alzheimer's and Parkinson's disease (Nikoletopoulou and Tavernarakis, 2012; Hernández et al., 2018).

Protein homeostasis disruption, implicated in several neurodegenerative diseases including ALS, involves two primary aspects: (1) the presence of misfolded protein aggregations requiring degradation, and (2) dysregulation of protein homeostasis, hindering neuronal ability to clear misfolded and aggregated proteins, which are pivotal in ALS pathology (Jeon et al., 2019).

Dysregulation of posttranscriptional RNA metabolism is involved in the pathophysiology of neurodegenerative diseases such as ALS/FTD (Jung et al., 2013). Stress alters mRNA translation through stress granule (SG) formation, which inhibits the synthesis of non-housekeeping proteins while maintaining the intact synthesis of protective proteins (Mann et al., 2019; Wolozin and Ivanov, 2019; Brown et al., 2020). SGs are transient, and dynamic membraneless organelles containing non-translating mRNAs and RNA-binding proteins (RBPs) that rapidly form and disperse through liquid–liquid phase separation (LLPS) in response to acute cellular stress (physiological SGs). In neurodegenerative disorders such as ALS, SGs can mature into persistent, non-dynamic pathological forms under chronic stress, acting as a nidus for disease-related protein aggregation (Khalfallah et al., 2018; Wolozin and Ivanov, 2019). Thus, targeting excitotoxicity, pathological SG formation, and protein homeostasis dysregulation represents therapeutic targets for ALS and other related neurodegenerative diseases.

Retinoic acid (RA), the active metabolite of vitamin A, acts via the retinoic acid receptors (RARs) to regulate the expression of a large number of genes (Mey and McCaffery, 2004). In the CNS, RA is crucial for the support of motor neurons and neuromuscular junction function; deficiency in animal models leads to motor neuron death, loss of coordination, and eventual paralysis (Corcoran et al., 2002).

ALS is associated with altered expression of genes and proteins in RA signalling pathways, including reduced levels of RARα and the RA-synthesising enzyme, retinaldehyde dehydrogenase (RALDH), in motor neurons (Corcoran et al., 2002; Riancho et al., 2016). Downregulation of genes encoding RAR-γ1 and the cytoplasmic RA carrier protein, cellular RA binding protein 1 (CRABP1), is also observed in the ventral horns and motor neurons of ALS patients (Jiang et al., 2005). Laser-captured microdissection concomitant with microarray technology showed decreased levels of CRABP1, RARα, and RARγ1 in ALS (Jiang et al., 2005). An inverse association has been noted between serum retinol-binding protein 4 (RBP4), which carries vitamin A throughout the body including the CNS, and the risk of ALS (Rosenbohm et al., 2018). These findings suggest a decline in endogenous RA signalling in ALS.

However, contrasting findings include elevated levels of RARβ and RXRβ in the cytoplasm of motor neurons of the SOD1G93A mutant rats (Jokic et al., 2007) and elevation of nuclear localisation of RARβ in motor neurons of spinal cord tissue from post-mortem ALS patients (Kolarcik and Bowser, 2012). Elevated RARβ levels in motor neurons were associated with reduced apoptosis markers, indicating a potential neuroprotective role (Kolarcik and Bowser, 2012); moreover, RARβ agonist enhanced motor neuron survival in primary motor neurons exposed to oxidative stress (Medina et al., 2020).

Therefore, the upregulation in RA signalling properties may represent a later compensatory response to an initial decline in RA signalling, promoting survival and regeneration. Alternatively, this increase may indicate dysregulation of RA signalling pathways, with receptors appearing at inappropriate times or locations, potentially harming the CNS.

Given the multifactorial nature of ALS and the potential of RA signalling pathways to offer protection against these factors, we investigated this in primary neuronal cultures and cell lines.

The study investigated a new series of high-affinity agonists for RARs, focussing on ellorarxine (also known as DC645 or NVG0645) (Khatib et al., 2020), which holds promise for treating various neurodegenerative diseases.

## Methods

### Animals

All animals were purchased from an in-house breeding colony of C57BL/6 mice in the University of Aberdeen animal facility. The animals were used in accordance with the EU Directive 63/2010EC and UK Home Office regulations. The experiments complied with the Animals (Scientific Procedures) Act 1986, amended in 2012.

### Cells

Neuroblastoma-spinal cord-34 (NSC-34) cells are an immortalised hybrid cell line produced from motor neuron–enriched embryonic mouse day 12–14 spinal cord cells fused with mouse aminopterin-sensitive neuroblastoma N18TG2 cells (Cashman et al., 1992). This cell line was generously provided by Dame Prof. Pamela J. Shaw (University of Sheffield, UK). The cells were cultured and maintained in DMEM with 4,500 mg/L glucose and L-glutamine supplemented with 10% FBS and 1% Penicillin–Streptomycin (Pen/Strep) in a tissue culture incubator with 5% CO_2_ at 37°C.

### Culture of primary cortical neurons

Brains were dissected sterilely from P0-P1 rat pups on ice in Gibco™ Neurobasal™ medium (Fisher Scientific). The forebrain was removed, and the olfactory bulbs, hippocampus, thalamus, and striatum were dissected away along with the meninges. The medium was carefully removed, and 500 μL per half cortex of the cortical digestion solution (Worthington Papain, UK Lorne Laboratories Limited) was added to the cortical pieces. They were gently triturated with a 1,000 μL tip 2–3 times. The homogenised tissue in the digestion solution was incubated in a tissue culture incubator with 5% CO_2_ at 37°C for 30 min, shaken every 10 min.

The digestion solution was then removed and replaced with the same volume of 1 mg/mL Gibco™ Soybean Trypsin Inhibitor (Fisher Scientific) or foetal bovine serum (VWR) to stop the digestion process, and the tube was incubated for 5 min at room temperature. The trypsin inhibitor solution was replaced with 3 mL Gibco™ Neurobasal™ medium (Fisher Scientific) supplemented with 2% serum-free Gibco™ B-27™ (Fisher Scientific), 1% Gibco™ GlutaMAX (Fisher Scientific), and 1% Pen/Strep, and the solution was triturated with the 1,000 μL tip 5 times, passed through a sterile cell strainer with a 70 μm nylon mesh (Fisher Scientific), and centrifuged at 1,400 *g* for 10 min. The supernatant was removed, and the pellet was reconstituted in 1 mL of neurobasal medium supplemented with 2% serum-free Gibco™ B-27 supplement 50 × (Fisher Scientific), 1% Gibco™ GlutaMAX (Fisher Scientific), and 1% Pen/Strep (Fisher Scientific). The cells were then counted and seeded on the 0.005% Poly-d-lysine (PDL)-coated glass coverslips at a density of 7 × 10^4^ cells per well. The 12-well plates were incubated in a tissue culture incubator with 5% CO_2_ at 37°C. Half the medium was changed every 3–5 days by gently removing the medium from the edge of the well and adding fresh medium without disturbing cells.

### Excitotoxicity procedure

Treatments to induce excitotoxic neuronal death were performed on rat primary cortical cell cultures grown on PDL-coated glass coverslips for 14 days *in vitro* (DIV14). A day before treatment, cells were treated with either 10 μM or 10 nM of ellorarxine, or with a DMSO control (concentration based on the highest percentage of DMSO used to ensure ellorarxine solubility, which was 0.01%). On the day of the experiment, prior to glutamate treatment, the medium was collected from each well and saved for later use.

Cells were rinsed with Gibco™ HBSS containing calcium and magnesium ([Ca^2+^]:1.26 mM & [Mg^2+^]: 0.95 mM; Fisher Scientific) supplemented with 4.2 mM NaHCO_3_ (Fisher Scientific), 10 mM HEPES (Fisher Scientific), and 35 mM D-glucose (Sigma-Aldrich). Control cells were then exposed to an HBSS-supplemented medium with DMSO. Experimental cells were exposed to HBSS-supplemented medium plus 100 μM glutamate, 10 μM co-agonist glycine, and either 10 μM or 10 nM of ellorarxine. The cells were incubated for 20 min in a tissue culture incubator with 5% CO_2_ at 37°C.

Immediately at the end of glutamate exposure time, cells were rinsed again with HBSS-supplemented medium, and the saved culture medium (collected before starting the glutamate assay) was replaced in each well. The cells were incubated in a tissue culture incubator with 5% CO_2_ at 37°C overnight. The cells were treated with ellorarxine (experimental) or DMSO (control) 24 h before glutamate exposure, during glutamate exposure, and 24 h after medium refreshment with preserved medium.

Finally, the cells were fixed in 4% PFA and used for immunocytochemistry (ICC) using MAP2 antibody as a neuronal marker and cleaved caspase-3 antibody as an indicator of neuronal apoptosis. Images were taken 24 h after refreshing the medium following glutamate treatment and compared to the relevant control of no glutamate exposure. Cell viability was determined by counting the number of MAP2-positive cells (live neurons), blind to each coded slide, using ImageJ software and manual counting.

The action of ellorarxine in reducing programmed cell death was studied using Western blotting to quantitatively determine changes in caspase-3 activation. The apoptosis pathway involves the activation of caspase-3 by cleavage, which increases cleaved caspase-3 and subsequently promotes cell death.

### MTT viability assay

MTT (3-(4,5-dimethylthiazol-2-yl)-2,5- diphenyltetrazolium bromide, Fisher Scientific) assay was used to assess cell number and viability. Mitochondrial reduction of MTT produces purple formazan crystals, the absorbance of which is quantified at 540 nm. The reduction occurs only when the mitochondrial reducing enzymes are active.

NSC-34 cells were grown in a 96-well plate until they reached 90% confluency. After appropriate treatment, 10 μl of a 5 mg/mL MTT solution was added to each well (final concentration: 0.5 mg/mL), the plate was wrapped in aluminium foil, and incubated for 2 h in a tissue culture incubator with 5% CO_2_ at 37°C. This incubation time was chosen based on optimal formazan crystal formation.

The supernatant was then removed, and 100 μl of DMSO was added to each well. The plate was shaken on an orbital shaker for 30 min at room temperature to dissolve the formazan crystals in DMSO. The absorbance was then read at 570 nm, with background absorbance at 690 nm subtracted.

### Immunocytochemistry

Cells were grown on PDL-coated glass coverslips in 6- or 12-well plates and fixed with 4% paraformaldehyde for 20 min. After washing, cells were blocked with blocking solution (10% serum solution, 0.1% Triton X-100 in phosphate-buffered saline) plus 0.3 M glycine.

Cells were incubated overnight at 4°C with primary antibodies in the blocking solution. The following primary antibodies were used: (1) microtubule-associated protein 2 (MAP2) present in neuronal soma and dendrite (Proteintech, 1:1,000); (2) cleaved caspase-3, as a neuronal apoptosis indicator (Cell signalling, 1:400); and (3) Ras GTPase-activating protein-binding protein 1 (G3BP1), as an SGs marker (Proteintech, 1:400).

The next day, cells were washed and incubated in secondary antibodies diluted in the blocking solution for 2 h at room temperature, protected from light. After incubation and washing three times, coverslips were added with a mounting medium containing bisbenzimide to label nuclei. The cells were imaged under a fluorescence microscope (Nikon Eclipse E400) or Zeiss confocal Airyscan 880 microscope.

### Sodium dodecyl sulphate polyacrylamide gel electrophoresis and western blotting assay

For immune immunoblot analysis of cleaved caspase-3, cells were grown in 6- or 12-well plates and subjected to an excitotoxicity assay. Following the assay, cells were gently washed with ice-cold 1 × PBS and lysed by ice-cold lysis buffer (150 mM NaCl, 50 mM HEPES, 1% Triton X-100, 10 μL/mL Halt™ Protease and Phosphatase Inhibitor Cocktail, EDTA-Free). Then, cells were scraped, passed through a 26-gauge needle, and centrifuged at 14,000 *g* for 20 min at 4°C.

Protein concentration was determined using the Thermo Scientific™ micro-BCA protein assay kit and measured with an Emax Precision Microplate Reader (Molecular Devices) at 560 nm. Equal amounts of protein (25 μg per lane) were loaded and separated on 4–12% SDS-PAGE gels, then transferred to nitrocellulose membranes using the Mini Trans-Blot Electrophoretic Transfer Cell system (Bio-Rad). Successful protein transfer and equal loading were verified by Ponceau S staining (0.1% w/v in 5% acetic acid), followed by washing with TBS-T.

The membrane was blocked for 1 h at room temperature in blocking buffer (10 mM Tris-HCl pH 7.5, 100 mM NaCl, 0.1% Tween 20) with 5% non-fat dry milk, then incubated overnight at 4°C with primary antibodies: Monoclonal cleaved caspase-3 (Cell Signalling, 1:1,000 dilution) and HRP-conjugated β-Actin (Proteintech, 1:3,000 dilution). Membranes were washed three times with TBS-T, then incubated for 1 h at room temperature with the appropriate HRP-conjugated secondary antibody in the blocking buffer. After three additional TBS-T washes, blots were developed using the Merck Millipore Immobilon™ Western enhanced chemiluminescence HRP detection kit. Cleaved caspase-3 protein levels were analysed and quantified densitometrically using the iBright FL1500 Western Blot Imaging System. Relative levels of cleaved caspase-3 were normalised to β-Actin, with results expressed as arbitrary units (mean ± SEM).

### Proteasome inhibition assay

To inhibit proteasomes in NSC-34 cells, epoxomicin (Sigma-Aldrich), a selective inhibitor of the 26S/20S proteasome, was used. Epoxomicin irreversibly inhibits the chymotrypsin-like (CT-L), trypsin-like and peptidyl-glutamyl peptide hydrolysing activities of the 26S/20S proteasome without affecting non-proteasomal protease activities such as trypsin (Cheng et al., 2011).

The NSC-34 cells were plated in a 96-well plate. At 80–90% confluency, the cells were pre-treated with 100 nM ellorarxine, 500 nM all-*trans* retinoic acid (atRA or RA), or DMSO (control) for 24 h. The following day, the cells were treated with or without 100 nM epoxomicin for 24 h before assessing survival using the MTT assay.

### Sodium arsenite treatment

NSC-34 cells were plated on 0.2% gelatin-coated glass coverslips in 12- and 24-well plates. When cells reached 60–70% confluency, they were pre-treated with either 10 μM or 10 nM of RAR ligand or DMSO (as control, ensuring solubility of ellorarxine which was a concentration of 0.01%) for 24 h prior to the experiment. The next day, cells were treated with 0.25 mM sodium arsenite (Sigma-Aldrich) for 45 min to induce stress granule formation, without changing the medium.

After treatment, cells on coverslips were washed with PBS, fixed with 4% PFA for 20 min, and subjected to immunocytochemistry (ICC) using G3BP1 antibody (Proteintech) as a stress granule-specific marker. The number of cells containing at least one stress granule was counted blindly using ImageJ, and the percentage of cells with SGs was calculated. Stress granules appeared either as single globular granules or cluster-like structures in the cytoplasm, some of which progressed to aggregated and non-dynamic (pathological) SGs. It was noted that some cells exhibited nuclear stress granules. The average sizes of SGs (with clustered granules considered as a single entity) were assessed using ImageJ software.

### Measurement of RAR ligands in tissue

The method utilised a previously developed technique for sensitive detection of RAR ligands (Kouchmeshky et al., 2020). This involved using F9-teratocarcinoma-derived Sil-15 reporter cells (F9-RARE-lacZ reporter cells), graciously provided by Dr. Michael Wagner (SUNY Downstate Medical Center, NY), to detect RAR ligands. These cells were cultured on a 0.2% gelatin-coated tissue culture-treated polystyrene surface in Dulbecco's Modified Eagle's Medium mixed with Ham's F-12 (1:1) (DMEM/F-12) supplemented with 1% GlutaMAX, 10% foetal bovine serum (FBS), and G-418 antibiotic for selection of the cells carrying the β-galactosidase (lacZ) gene.

Briefly, ellorarxine was administered intraperitoneally to mice at 1 mg/kg, and tissue distribution was examined 4 h post-injection. Euthanasia was performed using a carbon dioxide (CO_2_) chamber followed by cervical dislocation. Specific brain subregions and other tissues of interest were rapidly dissected, snap-frozen with dry-ice, and kept at −70°C until the experiment day. Brain regions included the rostral cortex (encompassing frontal association and primary and secondary motor areas) and the caudal lateral cortical region (comprising auditory cortex, temporal association areas, ectorhinal, and perirhinal areas) (Kirkcaldie, 2012).

Frozen tissues in tubes were rapidly weighed to prevent thawing, followed by homogenisation in a 2:1 ethanol/isopropanol lipid extraction solvent. After centrifugation to remove insoluble particles, the supernatant was added to 96-well plates containing the reporter cells at a concentration not exceeding 2% to avoid toxicity. Each sample was treated in triplicate and was incubated overnight in a tissue culture incubator with 5% CO_2_ at 37°C.

A standard curve was generated using ellorarxine concentrations ranging from 5 × 10^−4^ to 5 × 10^−11^ M applied to the cells. To account for any potential effect of brain lipid extracts, supernatant from non-injected homogenised brain samples was also included. Triplicate blank wells containing only the supernatant without RAR ligands were used to subtract background influence from other brain lipids.

The next day, cells were fixed, β-galactosidase reporter expression was determined by adding X-gal development solution and incubated at 37°C for up to 24 h, depending on signal intensity. The microplate was read at 615 nm on a microplate reader for colorimetric detection.

### qPCR analysis of transcript from mouse tissue

Mice were injected with 0.02 or 0.01 mg/kg ellorarxine, 2 days a week for 4 weeks. The injection solution of ellorarxine was freshly prepared in sterile 1 × PBS immediately prior to injection. Respective control mice received a maximum of 5% DMSO in 1 × PBS (chosen based on the percentage of DMSO used as a drug vehicle for injection into the experimental mice). The day after the last injection, the mice were euthanised using CO_2_ followed by cervical dislocation, and tissues of interest were snap-frozen immediately and stored at −70°C until the day of the experiment.

RNA extraction from tissue for quantitative polymerase chain reaction (qPCR) analysis was performed using Qiagen RNeasy mini kits (Qiagen) according to the manufacturer's protocols. Tissue samples (up to 20 mg) were homogenised with a Pestle Motor Mixer in the kit lysis buffer and further triturated through a 26-gauge needle. On-column DNase digestion was performed using a RNase-Free DNase Set kit (Qiagen). RNA concentration of each sample was measured using a NanoDrop™ 2000c spectrophotometer (Thermo Fisher Scientific). Primers were designed using Primer-BLAST (see Table 1 for the list of sequences). cDNA was synthesised by reverse transcription using qScript cDNA SuperMix (Quantabio), and qPCR reactions were performed using SYBR-Green (Takyon No ROX SYBR 2X MasterMix blue dTTP), following the manufacturer's instructions. Negative controls included minus RT-control reactions for the primer pair.

**Table 1:** List of primers designed for Mus musculus mRNA amplification and used for the evaluation of the levels of gene induction following the administration of low-dose DC645 in mice.

**Name**	**Forward primer sequence (5^′^ → 3^′^)**	**Reverse primer sequence (5^′^ → 3^′^)**
*Rbp4*	TCAAGATGAAGTACTGGGGTGTAG	GAGAAAACACAAAGGAGTAGCTGT
*Rarb*	CCAAGTGCATTATTAAGATCGTGGA	TTTAGTGTAAGGCCATCAGAGAAAG
*Cyp26b1*	GAGACTGGTCACTGGTTGCTA	GTCTTGAAAACGTTGCCATACTTC
*Aldh1a1*	TAACTGCTATATGATGTTGTCAGCC	GAGATATCTTCATTGCGACTGTCTT
*Tnfa*	GTCTACTGAACTTCGGGGTGA	CTGATGAGAGGGAGGCCATTT
*Il1b*	CACCTTTTGACAGTGATGAGAATGA	GAGATTTGAAGCTGGATGCTCTC
*Igf1*	GATGCTCTTCAGTTCGTGTGT	ACAGTACATCTCCAGTCTCCTC
*Gapdh*	GTCCCGTAGACAAAATGGTGAAG	GAACATGTAGACCATGTAGTTGAGG
*Actb*	GATCAAGATCATTGCTCCTCCTG	GGTGTAAAACGCAGCTCAGTAA

A Roche LightCycler 480 real-time thermocycler was used with the following programme: initial Takyon™ activation at 95°C for 3 min, followed by 40 cycles of denaturation at 95°C for 10 s, annealing at 60°C for 60 s, and extension at 60°C for 40 s. Subsequently, a melting curve was obtained by heating the plate at 95°C for 5 s followed by 58°C for 1 min. Melt curve analysis was performed to assess contamination using Light Cycler 480 1.5 software. The standard curve was evaluated for linearity, with efficiency considered acceptable between 1.8 and 2. The 2^−Δ*ΔCT*^ method (Livak and Schmittgen, 2001) was employed to analyse the relative changes in gene expression normalised to the geomean of CT values of two housekeeping genes, Actin β and *Gapdh*, as the reference genes.

### Statistical analysis

For primary and cell line cultures, three to four independent cell cultures with their respective controls were used. Data from each cell line (*n* = 3–4 independent passages) were presented as arithmetic means of replicates, with statistical significance determined using Prism software (version 10). Statistical comparisons between control and experimental groups was performed by one-way analysis of variance (ANOVA) followed by Tukey's multiple comparisons *post-hoc* test for comparisons among all groups showing significance, and Dunnett's *post-hoc* test for comparisons against a single control group, where appropriate. A significance level of *P* < 0.05 was considered statistically significant.

The number of animals per experiment was determined by prior power analysis to provide sufficient statistical power. For dose–response and RA bioassay experiments, a minimum sample size of six mice per treatment group was used, based on an 80% statistical power level, accepting *p* < 0.05, and anticipating a variability in observation of 35%. A change of 45% or more was considered of interest.

## Results

### Neuronal protection by RAR agonist ellorarxine for glutamate induced excitotoxicity of rat neuron-glia cortical co-cultures

Excitotoxicity, a mechanism implicated in neuronal death across various types of ALS (Bacman et al., 2006; Nikoletopoulou and Tavernarakis, 2012; King et al., 2016), represents a target for therapeutic intervention with limited success to date.

**Figure 1:**
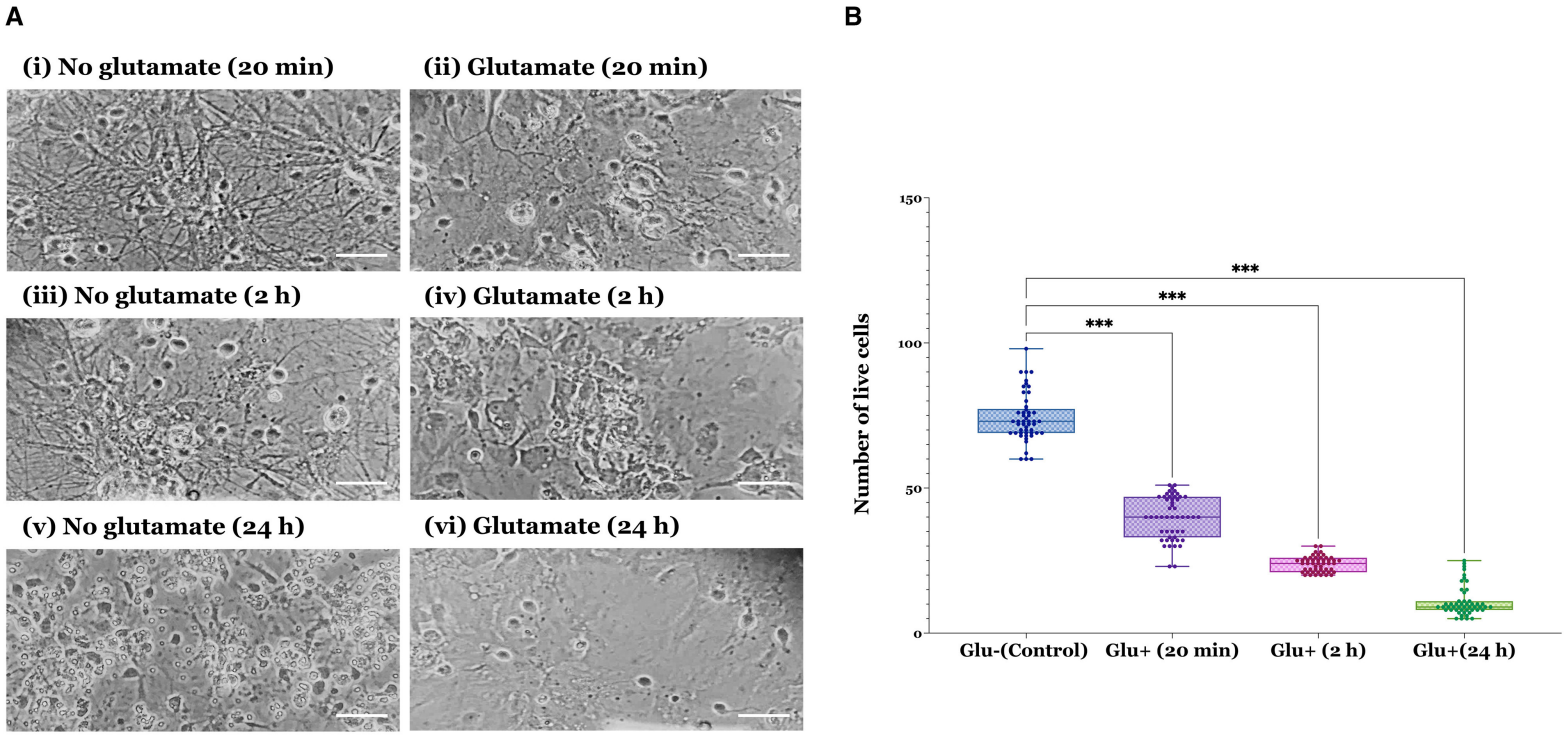
Verification of glutamate excitotoxicity assay in rat cortical neuron-glia co-cultures across different durations of glutamate exposure. **(A)** Cells were exposed to 100 μM glutamate (and 10 μM glycine as a co-agonist) at DIV14 for varying durations before refreshing the medium and incubating for an additional 24 h. (i) 20 min control (no glutamate), (ii) 20 min glutamate, (iii) 2 h control (no glutamate), (iv) 2 h glutamate, (v) 24 h control (no glutamate), and (vi) 24 h glutamate. Images show increasing deterioration and apparent cell loss over time (scale bar: 30 μm). **(B)** The number of live cells significantly decreased under excitotoxic conditions. Data represent the mean values of live cells from four independent biological replicates, with 50 observations per group, totalling 200 observations across four groups. The bar heights represent the mean, and error bars show the range from minimum to maximum values (whiskers), with all individual data points overlaid. Statistical significance was determined by one-way ANOVA [*F*_(3, 196)_ = 760.6, *P* < 0.001], and pairwise comparisons between control and treatment groups were conducted using Dunnett's multiple comparison *post-hoc* test (***P < 0.001).

In this study, we investigated the potential neuroprotective effects of RAR ligand ellorarxine in an excitotoxicity assay using primary mixed cortical neurons-glia cultures. Initially, the assay was optimised using cultures at DIV14, assessing the impact of different durations (20 min, 2 h, and 24 h) of exposure to 100 μM glutamate and 10 μM glycine. Images taken 24 h post-glutamate exposure revealed a progressive loss of cells with increasing exposure time: from acute (20 min) exposure (Figure 1A-ii) to 2 h (Figure 1A-iv), culminating in substantial cell loss after 24 h (Figure 1A-vii). Figure 1A-i, iii, and v show controls (no glutamate) of each treatment, respectively. Based on these observations and quantifications, which showed a significant decrease in the number of live cells post-glutamate exposure over time (Figure 1B), a 20-min exposure to glutamate was selected for subsequent ellorarxine neuroprotection assays.

**Figure 2:**
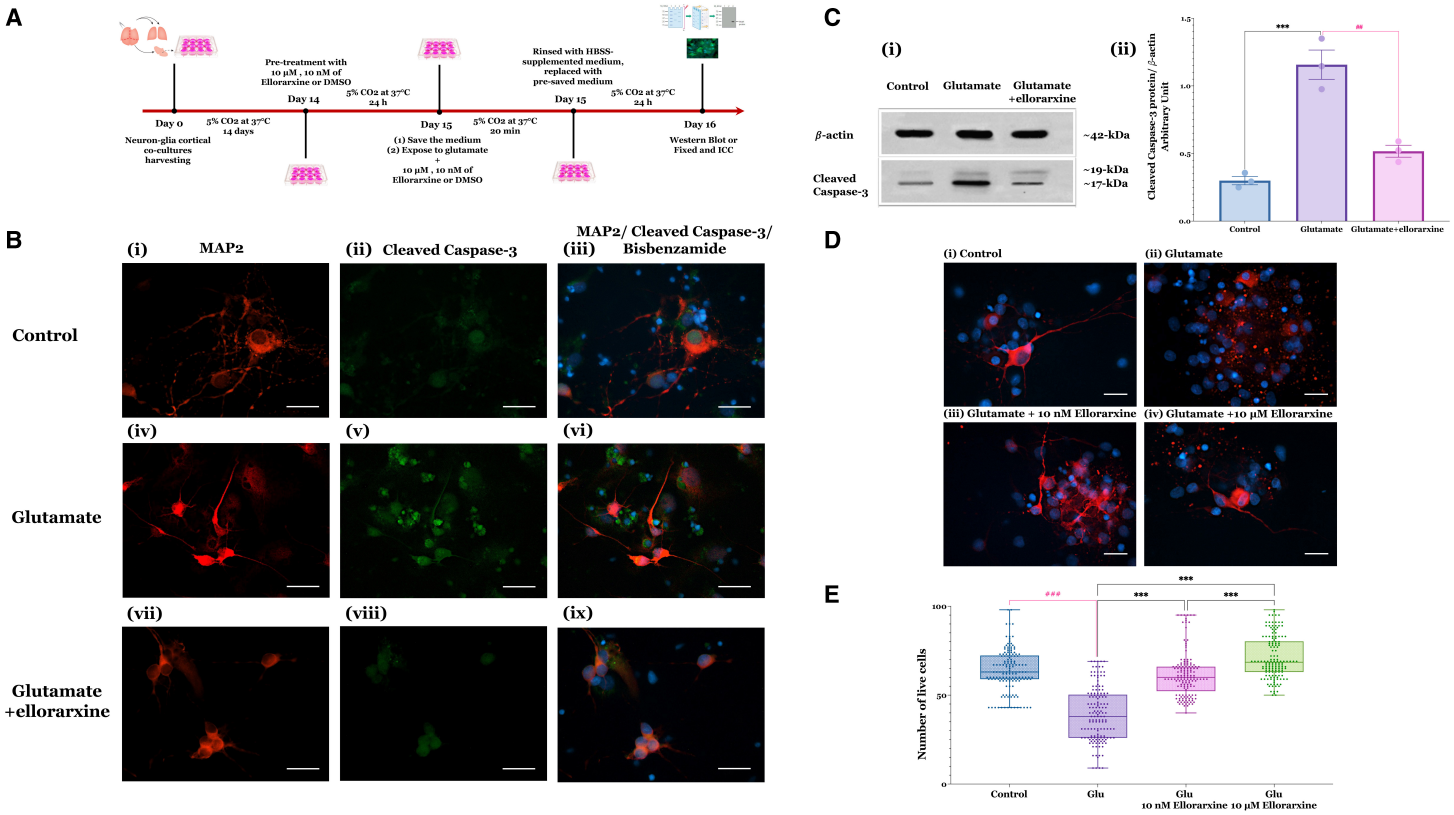
Glutamate-induced excitotoxicity in neuron-glia co-cultures and protective effects of ellorarxine. **(A)** Timeline of the excitotoxicity experiments to study the effects of ellorarxine. **(B)** Primary neuron-glia cultures (DIV14) were either untreated or pre-treated with DMSO (vehicle) or 10 μM ellorarxine for 24 h before exposure to 100 μM glutamate (and 10 μM glycine) for 20 min. Following overnight incubation with pre-reserved medium, cells were fixed and subjected to double ICC using antibodies to MAP2 (a neuronal dendrite marker, red) and cleaved caspase-3 (an indicator of neuronal apoptosis, green), with bisbenzimide counterstained (nuclear marker, blue). Panels show (i–iii) control (DMSO) without ellorarxine or glutamate, (iv–vi) cells exposed to glutamate for 20 min, and (vii–ix) ellorarxine pre-treated cells before glutamate exposure. Glutamate treatment increased the number of apoptotic cells, indicated by increased cleaved caspase-3 labelling, which decreased with ellorarxine treatment. The cleaved caspase-3 labelling specifically highlights the pathway of cell death following glutamate exposure. **(C)** (i) A western blot analysis of cleaved caspase-3 activation in cells exposed to glutamate, with and without ellorarxine pre-treatment, was performed using a cleaved-caspase-3 antibody as an apoptosis marker and β-actin as a housekeeping protein for normalisation. (ii) Quantification of western blot results showed significant upregulation of cleaved caspase-3 activation after exposure to glutamate. This upregulation was significantly reduced to levels near the control in cells pre-treated with ellorarxine before glutamate exposure. Data are plotted as mean values of activated caspase-3 from three independent biological experiments. Error bars represent SEMs. Statistically significant differences were determined by one-way ANOVA [*F*_(2, 6)_ = 40.72, *P* < 0.001], and pairwise comparisons between control and treatments were performed using Tukey's multiple comparison *post-hoc* test (***P < 0.001, ^##^*P* < 0.01) (for the densitometric quantification of cleaved caspase-3, both the 17 and 19 kD bands were measured together). **(D)** ICC for MAP2 (red) with bisbenzimide counterstaine (blue) to quantify neuronal loss, showing (i) control (DMSO) without ellorarxine and glutamate, (ii) glutamate exposure for 20 min, and pre-treatment with (iii) 10 μM or (iv) 10 nM ellorarxine before glutamate exposure. **(E)** The number of MAP2-positive neurons (live neurons) were counted blindly. Both 10 μM or 10 nM ellorarxine significantly increased the number of neurons under excitotoxic conditions. Data was presented as the mean values of MAP2-positive neurons from three independent biological replicates, each analysed in duplicate. In total, around 150 microscopic fields were counted per condition to obtain an accurate and comprehensive estimate. The bar heights represent the mean, and error bars show the range from minimum to maximum values (whiskers), with all individual data points overlaid. Statistically significant differences were determined by one-way ANOVA [*F*_(3, 596)_ = 122.2, *P* < 0.001], and pairwise comparisons between control and treatments were carried out using Turkey's multiple comparison *post-hoc* test (***P < 0.001, ^###^*P* < 0.001).

To assess ellorarxine's neuroprotective potential, DIV14 rat cortical neuron-glia co-cultures were pre-treated with either 10 μM or 10 nM ellorarxine, or DMSO (control) for 24 h. This was followed by a 20-min exposure to 100 μM glutamate and 10 μM glycine or HBSS (control). After glutamate exposure, cells were rinsed with an HBSS-supplemented medium, refreshed with a pre-saved culture medium, and incubated for 24 h. In the treatment groups, ellorarxine was administered pre-, during, and post-exposure to glutamate. Subsequently, cells were fixed and subjected to double ICC to label neurons with MAP2 and assess cleaved caspase-3 activation as an indicator of neuronal apoptosis (Figure 2A).

Qualitative analysis appeared to show a reduction in cleaved caspase-3 labelling in cells treated with 10 μM ellorarxine (Figure 2B). Western blotting quantification of caspase-3 cleavage revealed that glutamate significantly increased cleaved caspase-3 activation, which was notably reduced to near-control levels by ellorarxine pre-treatment (Figure 2C-i, Cii). Quantification of MAP2-positive neurons demonstrated a significant increase in live cells in cultures pre-treated with either 10 μM or 10 nM ellorarxine compared to control (DMSO) under excitotoxic conditions (Figures 2D, E).

### RAR agonist ellorarxine pre-treatment protects NSC-34 cells against proteasome inhibition-induced cell death

Decline in proteasome function has been proposed as one mechanism of cell death in ALS, given the presence of inclusion bodies containing aggregated protein in the disease (Jeon et al., 2019; Lambert-Smith et al., 2022). To investigate this, an ALS model in NSC-34 cells was established by inhibiting proteasome activity with 100 nM epoxomicin. Epoxomicin is a potent and selective irreversible inhibitor of chymotrypsin-like, trypsin-like, and peptidyl-glutamyl peptide hydrolysing catalytic activities of the 26S/20S proteasome, without affecting the activities of non-proteasomal proteases such as trypsin, chymotrypsin, and cathepsin B (Meng et al., 1999; Cheng et al., 2013).

**Figure 3:**
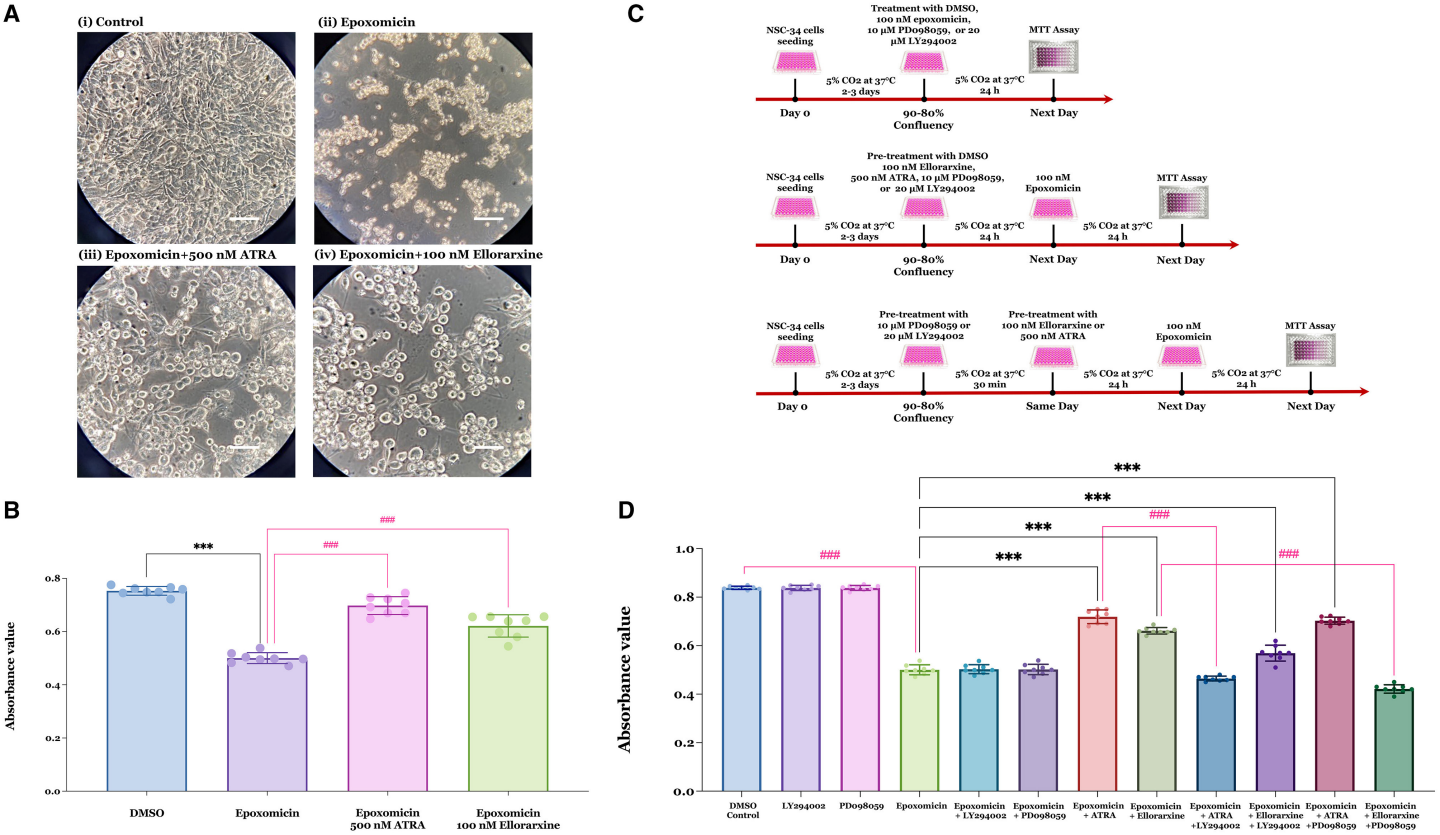
Neuroprotective effects of RAR ligands against epoxomicin-induced apoptosis and potential kinase-mediated pathways of action. **(A)** The viability of NSC-34 cells was assessed following treatments with either (i) DMSO (control), (ii) DMSO pre-treatment for 24 h followed by 100 nM epoxomicin (a proteasome inhibitor), (iii) 500 nM atRA pre-treatment for 24 h followed by epoxomicin, and (iv) 100 nM ellorarxine pre-treatment for 24 h followed by epoxomicin. The number of viable cells appeared to reduce after epoxomicin treatment compared to the DMSO control. However, cells pre-treated with atRA or ellorarxine before epoxomicin treatment showed a lesser reduction in cell number. Light microscopy images were captured at 400 × magnification. **(B)** The number of live cells was quantified using an MTT assay. Both atRA and ellorarxine pre-treated cells exhibited significantly less decline in viability compared to cells treated with epoxomicin alone. Data were plotted as mean values of the number of live cells (based on absorbance) from four biological replicates (independent experiments), each analysed in duplicate. Error bars represent SEMs. Statistically significant differences were determined by one-way ANOVA [*F*_(3, 28)_ = 107.2, *P* < 0.001], and pairwise comparisons between control and treatments were performed using Tukey's multiple comparison *post-hoc* test (***P < 0.001, ^###^*P* < 0.001). **(C)** Time-line of experiments determining epoxomicin-induced apoptosis, protection by RAR agonists and potential involvement of kinase-mediated pathways. **(D)** To evaluate the role of kinases in the neuroprotective effect of atRA and ellorarxine against epoxomicin-induced neurotoxicity, NSC-34 cells were treated with either DMSO (control) or DMSO for 24 h followed by 100 nM epoxomicin for 24 h. The protective effects of 500 nM atRA or 100 nM ellorarxine pre-treatment for 24 h, followed by 100 nM epoxomicin, were assessed. The involvement of kinases in this protective effect was examined using either 20 μM of the PI3/Akt inhibitor LY294002 or 10 μM of the MAPK/ERK1/2 inhibitor PD098059 for 30 min prior to the RAR ligands. Neither inhibitor showed toxicity or neuroprotection when applied alone or in combination with epoxomicin. LY294002, the PI3/Akt inhibitor, significantly reduced atRA's neuroprotection against epoxomicin-induced neurotoxicity but did not significantly affect ellorarxine's neuroprotection. Conversely, PD098059, the MAPK/ERK1/2 inhibitor, did not block atRA's neuroprotection but inhibited ellorarxine's neuroprotection. Data were plotted as mean values of the number of live cells (based on absorbance) from four biological independent experiments, each analysed in duplicate. Error bars represent SEMs. Statistically significant differences were determined by one-way ANOVA [*F*_(11, 84)_ = 563.9, *P* < 0.001], and pairwise comparisons between control and treatments were performed using Tukey's multiple comparison *post-hoc* test (***P < 0.001, ^###^*P* < 0.001).

Treatment of NSC-34 cells with 100 nM epoxomicin for 24 h resulted in a reduction in cell number. However, pre-treatment with 500 nM atRA or 100 nM ellorarxine 24 h prior to epoxomicin exposure appeared to demonstrate neuroprotective activity (Figure 3A). Quantification by MTT assay after 24 h of epoxomicin exposure revealed that epoxomicin induced a 37% decrease in cell viability (Figure 3B). In contrast, pre-treatment with 500 nM atRA or 100 nM ellorarxine 24 h before epoxomicin exposure significantly increased the number of viable cells compared to those treated with epoxomicin (Figure 3B).

### Downstream signalling pathways associated with RAR ligand neuroprotection from epoxomicin-induced toxicity

Possible pathways by which RAR activation provides neuroprotection against epoxomicin-induced toxicity were investigated. Two principal pathways for neuronal survival are the extracellular signal-regulated kinase 1/2 (ERK) pathway, part of the mitogen-activated protein kinase (MAPK) family, and the phosphoinositide-3-kinase (PI3K) pathway, which activates the serine/threonine protein kinase B (PKB/Akt) (Xia et al., 1995; Dent, 2014). To assess whether either of these pathways was involved in the neuroprotective action of RAR agonist following epoxomicin treatment, selective inhibitors were used to determine if they blocked neuroprotection.

NSC-34 cells were treated with DMSO (control), 100 nM epoxomicin, 10 μM PD098059 (a MAPK/ERK1/2 inhibitor), or 20 μM LY294002 (a PI3/Akt inhibitor) alone for 24 h. Alternatively, cells were pre-treated with 10 μM PD098059, or 20 μM LY294002 for 30 min prior to exposure to 100 nM epoxomicin for 24 h. Additionally, cells were pre-treated with 100 nM ellorarxine or 500 nM atRA for 24 h, followed by 100 nM epoxomicin for 24 h. Finally, cells were pre-treated with 10 μM PD098059, or 20 μM LY294002 for 30 min prior to pre-treatment with 100 nM ellorarxine or 500 nM atRA for 24 h, followed by 100 nM epoxomicin for 24 h (Figure 3C). Then, cell viability was determined by MTT assay. Results showed that both atRA and ellorarxine provided neuroprotection against epoxomicin-induced neurotoxicity (Figure 3D). However, LY294002, the PI3/Akt inhibitor, completely blocked atRA's neuroprotective action against epoxomicin-induced neurotoxicity, while it only partially reduced ellorarxine's neuroprotective action (Figure 3D). In contrast, PD098059, the MAPK/ERK1/2 inhibitor, did not affect atRA's neuroprotection against epoxomicin-induced neurotoxicity but completely blocked ellorarxine's neuroprotective action (Figure 3D). This suggests that atRA and ellorarxine utilise different pathways for their neuroprotective effects.

Neither of these inhibitors showed toxicity when exposed to the cells alone for 24 h, nor did they affect epoxomicin-induced neurotoxicity when used in combination with epoxomicin (Figure 3D).

### The effect of RAR agonist ellorarxine on sodium arsenite-induced stress granule size and number in NSC-34 cells

A further route by which proteostasis may influence ALS is through its role in controlling the turnover of SGs (Wolozin and Ivanov, 2019; Hu et al., 2022). Stress granules are transient structures, and their assembly and disassembly are a normal part of the cell's response to stress. However, abnormal dynamics under conditions of chronic cellular stress may lead to enduring and larger structures, known as pathological SGs (Mann et al., 2019; Wolozin and Ivanov, 2019). This study investigated the influence of the RAR agonist ellorarxine on stress granule size and number.

**Figure 4:**
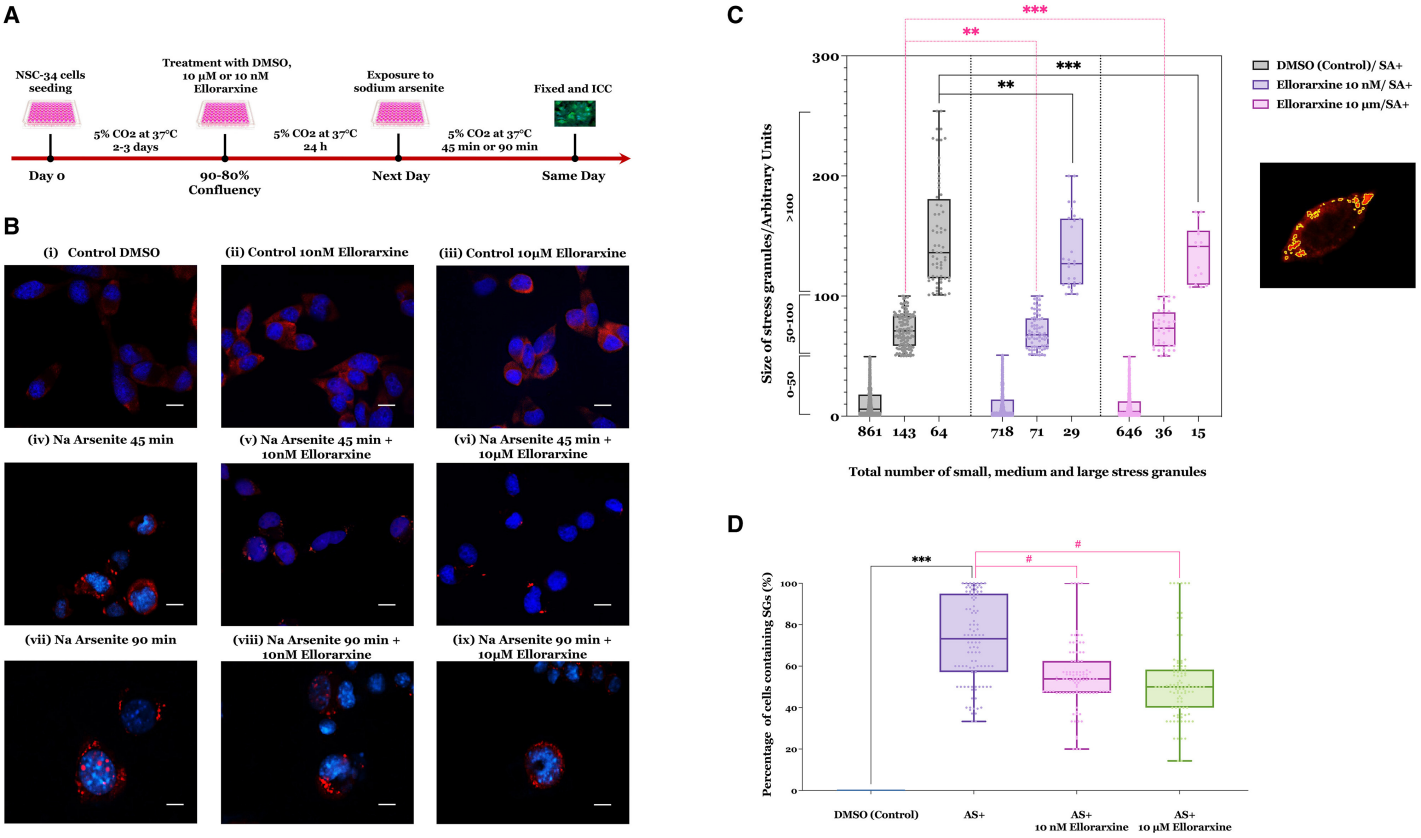
Effect of ellorarxine on number of small, medium, and large stress granules in NSC-34 cytoplasm following sodium arsenite exposure. **(A)** Timeline of the sodium arsenite-induced stress granules in NSC-34 cells and the effect of ellorarxine on the formation, number, and size of SGs. **(B)** NSC-34 cells were pre-treated with 10 nM or 10 μM ellorarxine for 24 h before exposure to 0.25 mM sodium arsenite (SA) for 45 min, followed by ICC for G3BP1. **(C)** The average sizes of stress granules (not distinguishing between large individual SGs or those that are clustered) were assessed using ImageJ software. In total, 150 cells from 20 randomly chosen microscopic fields were counted and analysed blindly for each of the three biologically independent experiments performed in duplicate. Pre-treatment of the cells with 10 μM ellorarxine before sodium arsenite exposure significantly reduced the number of stress granules in all three size categories (0–50, 50–100, and >100 nm^2^). The graph illustrates the distribution of data depicting the number of SGs (*x*-axis) across specific size ranges (*y*-axis) using a box and whisker plot. Each box plot displays the mean height, with error bars indicating the range from minimum to maximum size of SGs within each group, and the median represented by a horizontal line inside the box. Individual data points are overlaid on the plot to show the complete dataset distribution. Statistical significance was determined using one-way ANOVA [*F*_(2, 447)_ = 208.8, *P* < 0.001], followed by pairwise comparisons between the control and treatment groups using Dunnett's multiple comparison *post-hoc* test (**P < 0.01, ***P < 0.001). **(D)** The number of cells containing at least one stress granule was also counted after pre-treatment with either 10 μM ellorarxine or 10 nM ellorarxine followed by 45 min of 0.25 mM sodium arsenite exposure. This number was significantly lower than in cells treated only with sodium arsenite across three independent biological replicates examined in duplicate. The bar heights represent the mean, error bars show the range from minimum to maximum values (whiskers), and the middle line indicates the median, with all individual data points overlaid. Statistical significance was determined by one-way ANOVA [*F*_(3, 396)_ = 180.1, *P* < 0.001], with pairwise comparisons between cells exposed to SA alone and cells pre-treated with ellorarxine and exposed to SA using Turkey's multiple comparison *post-hoc* test (***P < 0.001, ^#^*P* < 0.05).

Stress granules assembly was induced in the NSC-34 cell line by treatment with sodium arsenite (0.25 mM) for 45 and 90 min. Sodium arsenite is an inducer of oxidative stress commonly used to induce SGs (Wang et al., 2012). The timeline of sodium arsenite-induced stress granule generation in the presence and absence of ellorarxine in NSC-34 cells is shown in Figure 4A. Stress granules were identified using Ras GTPase-activating protein-binding protein 1 (G3BP1) as a marker. No SGs were detected in the control group, and G3BP1 remained diffusely distributed in the cytoplasm. However, SGs were observed in almost all cells by 90 min exposure to sodium arsenite (Figure 4B).

Cells pre-treated with 10 nM or 10 μM ellorarxine 24 h before exposure to sodium arsenite for 45 or 90 min appeared to show both fewer and smaller SGs compared to the control (Figure 4B). The SGs either appeared as single globular granules or cluster-like globular structures in the cytoplasm. After 90 min of exposure to sodium arsenite, a greater number of cells showed SGs inside the cytoplasm, and some cells displayed nuclear SGs. In contrast, cells pre-treated with 10 μM ellorarxine appeared to have more diffuse and smaller SGs inside the cytoplasm.

When this was quantified (Figure 4C) pre-treatment of the cells with 10 μM ellorarxine before sodium arsenite exposure significantly reduced both the numbers and sizes of the SGs. Additionally, the number of cells containing at least one SG was significantly lower in cells pre-treated with either 10 μM or 10 nM ellorarxine 24 h before exposure to sodium arsenite for 45 min compared to DMSO-treated cells exposed to sodium arsenite (Figure 4D).

### Tissue distribution and induction of gene expression by ellorarxine after administration into mice

**Figure 5:**
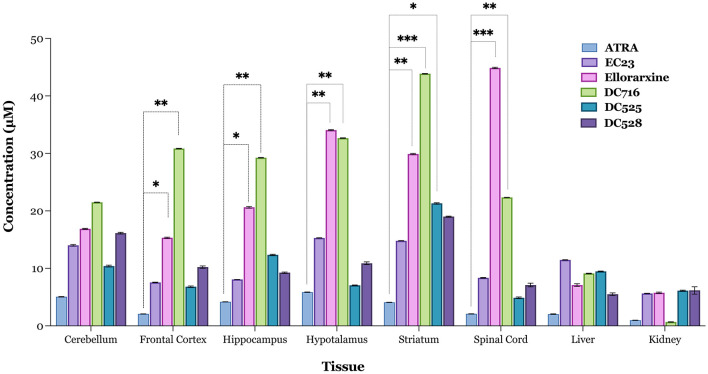
Tissue localisation of RAR ligands 4 h after intraperitoneal administration. Tissue concentrations of ellorarxine and other ligands for the RARs were determined using the Sil-15 reporter assay to quantitate the capacity of RAR ligand (atRA, EC23, ellorarxine, DC716, DC525, and DC528) to induce RAR-mediated transcription. Tissue lipid extracts were prepared after injection with 1 mg/kg RAR ligand or DMSO vehicle control. The results were plotted with control values subtracted, and data are presented as the mean values of the RAR ligand concentration in the tissue of six independent biological replicates analysed in triplicate. The error bars represent Standard Deviation (SD) and significance determined by one-way ANOVA [*F*_(5, 12)_, *P* < 0.001] with pairwise comparisons between control (endogenous RA) and treatments (RAR ligands) in each tissue carried out using the Dunnett's multiple comparison *post-hoc* test (*P < 0.05, **P < 0.01, ***P < 0.001).

The studies point to a series of potential neuroprotective effects of the RAR agonist ellorarxine *in vitro*. To explore these effects *in vivo*, the tissue distribution and gene expression induction of ellorarxine were examined following its administration into mice compared to other related RAR agonists (Kouchmeshky et al., 2020). Animals were injected intraperitoneally (ip), and the concentration of RAR agonists was determined after 4 h post-injection from lipid extracts of tissues using a reporter cell-based assay previously described (Kouchmeshky et al., 2020), and normalised to tissue weight. Ellorarxine and DC716 showed significant accumulation in the spinal cord, frontal cortex, hippocampus, striatum, and hypothalamus. Conversely, DC525 exhibited the lowest concentration of synthetic RAR ligands in the spinal cord (Figure 5).

The long-term influence of ellorarxine on gene expression in various tissues was investigated by examining the expression of several retinoid-regulated genes, including *Rbp4, Cyp26b1, Rarb*, and *Aldh1a1*, as reporters of RAR activation. Additionally, genes associated with RA- regulated neuroinflammation, such as *Tnfa* (tumour necrosis factor alpha) and *Il1b* (interleukin 1 beta), were analysed to determine if their levels decrease, along with the gene associated with RA-regulated growth, Igf1 (insulin-like growth factor 1) (Khatib et al., 2020).

**Figure 6:**
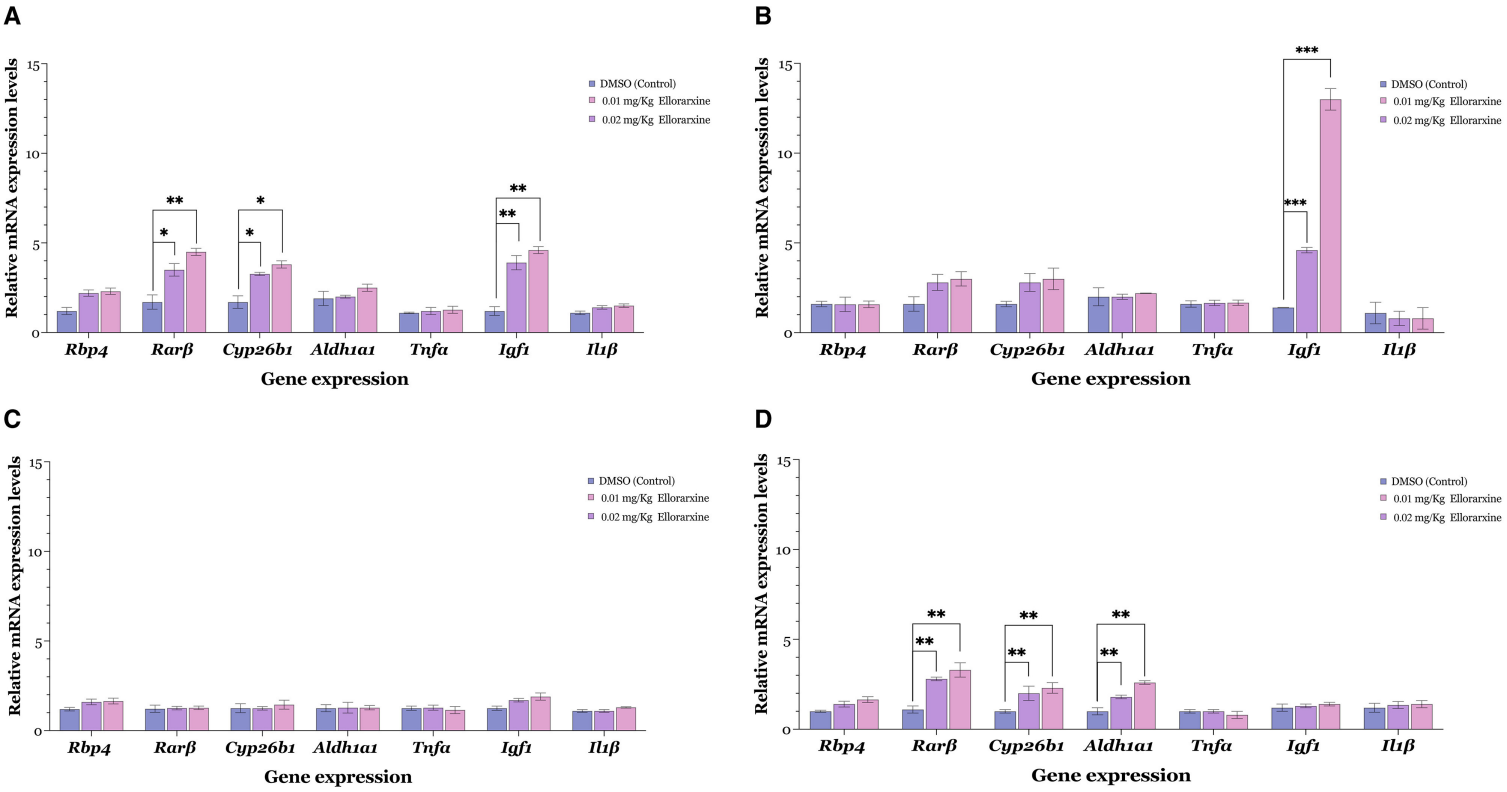
Gene induction in various mouse tissues following serial intraperitoneal injection of low dose ellorarxine. Mice were treated with 0.01 or 0.02 mg/kg ellorarxine 2 days a week for 4 weeks, or with DMSO vehicle as a control. Transcript levels of *Rar*β, *Cyp26b1*, and *Igf1* (*Gapdh* and *Actin* β used for normalisation) were quantified by qPCR in **(A)** spinal cord, **(B)** frontal cortex, **(C)** lateral caudal cortex, and **(D)** liver. Data are presented as mean values of the relative mRNA levels from six independent biological replicates analysed in triplicate. **(A)** Both doses of ellorarxine (0.02 and 0.01 mg/kg) significantly increased the expression levels of *Rar*β, *Cyp26b1*, and *Igf1* genes above control in the spinal cord. **(B)** In the frontal cortex, ellorarxine significantly increased the expression levels of the *Igf1* gene compared to the control. **(C)** In the lateral caudal cortex, ellorarxine did not induce significant changes in gene expression levels. **(D)** In the liver, both doses of ellorarxine significantly increased the expression levels of *Rar*β*, Cyp26b1*, and *Aldh1a1* genes compared to control. Error bars indicate SEMs and the statistical significance determined by one-way ANOVA [*F*_(2, 15)_, *P* < 0.001], with pairwise comparisons between control and treatments carried out using the Dunnett's multiple comparison *post-hoc* test (*P < 0.05, **P < 0.01, ***P < 0.001).

Ellorarxine at a dose of 0.01 or 0.02 mg/kg administered twice a week for 4 weeks significantly increased the expression levels of *Rarb, Cyp26b1*, and *Igf1* above control levels in the spinal cord (Figure 6A). In the frontal cortex, there was only a single gene change, marked by a significant increase in *Igf1* (Figure 6B). This contrasted with the lateral caudal cortex, where there were no changes in gene expression levels induced by ellorarxine implying intriguing region-specific effects of the compound (Figure 6C). In the liver (Figure 6D), both doses of ellorarxine (0.01 and 0.02 mg/kg) significantly increased the expression levels of *Rarb, Cyp26b1*, and *Aldh1a1*. However, ellorarxine did not significantly affect the levels of other genes above the controls.

## Discussion

Current clinical treatments for ALS, including riluzole, along with experimental treatments such as tofersen (an antisense oligonucleotide targeting SOD1 mRNA), edaravone (an antioxidant), and relyvrio (a stress signal blocker) have demonstrated limited efficacy in halting or slowing disease progression. This study highlights the potential neuroprotective effects of RAR agonists in several ALS-related cellular events and their specific localisation in disease-relevant sites. Initial assays focussed on neuronal excitotoxicity, a critical factor contributing to motor neuron degeneration in ALS (Foran and Trotti, 2009). Glycine was used in these assays combined with glutamate because NMDA is a Ca^2+^-permeable receptor plugged by Mg^2+^ and requires binding of glutamate and co-agonist glycine 10 μM to open and allow the influx of Ca^2+^, Na^+^, and K^+^ ions to conduct current.

The distinct characteristics of upper and lower motor neurons affected in ALS underscore their differential origins, locations of cell bodies, target tissues, neurotransmitter usage, and intracellular pathways (Zayia and Tadi, 2023). Upper motor neurons project from the cerebral motor cortex to the brainstem and spinal cord, predominantly utilising glutamate as a neurotransmitter, whereas lower motor neurons project from the brainstem and spinal cord to muscles and glands, primarily using acetylcholine (Stifani, 2014; Zayia and Tadi, 2023). To model glutamatergic excitotoxic damage, we employed cortical neurons known for their sensitivity to glutaminergic excitotoxic damage, while utilising NSC-34 cells as a lower motor neuron-like model in other assays. Importantly, NSC-34 cells, despite expressing glutamate receptor subunits, do not elicit a calcium influx in response to glutamate and are therefore unsuitable for excitotoxicity assays (Hounoum et al., 2016).

One proposed mechanism of excitotoxicity in ALS involves the excessive stimulation of glutamate receptors due to impaired clearance of glutamate from the synaptic cleft. This clearance is primarily regulated by the excitatory amino acid transporter 2 (EAAT2) expressed by astrocytes, and reductions in EAATs have been observed in a SOD1 mouse model of ALS as well as in the post-mortem cortical and spinal cord tissues of fALS and, to a greater extent, sALS patients (Rothstein et al., 1995; Trotti et al., 1999; Vucic and Kiernan, 2013; King et al., 2016). Elevated glutamate levels in the cerebrospinal fluid (CSF) of ALS patients suggest compromised glutamate reuptake by glial and neuronal cells (Bading, 2017). Ellorarxine may exert neuroprotective effects by enhancing the clearance of glutamate from the synaptic cleft. RA has been shown to upregulate EAAT2 expression in astrocytes through post-translational mechanisms, and this expression is also tightly controlled at the translational level (Tian et al., 2007; Colton et al., 2010). Our observation of massive cellular death in the pure neuronal culture compared to the mixed co-culture in the same assay condition (data was not shown) underscores the potential role of glia and specifically astrocyte, through EAAT2-mediated glutamate clearance, in regulating excitatory responses. Thus, ellorarxine's ability to significantly enhance cell viability under glutamate excitatory conditions may be attributed to its induction of EAAT2 levels. Further investigation is needed to elucidate the correlation between RAR agonists' neuroprotective effect in glutamate-induced excitotoxicity and astrocytic EAAT2 levels at post-translational levels.

Ellorarxine's neuroprotective action in glutamate-induced excitotoxicity involves additional mechanisms, particularly its modulation of two kinase systems. Glutamate typically inhibits the PI3K/Akt/GSK3b (phosphoinositide 3-kinases/protein kinase B/glycogen synthase kinase-3 beta) survival pathway while activating JNK/P38-MAPK (c-Jun N-terminal kinases/p38 mitogen-activated protein kinase), leading to mitochondrial apoptosis. RA has been shown to enhance the PI3K/Akt pathway (Pein et al., 2017) both in long-term via genomic mechanisms and short-term through a non-genomic route, and it can also inhibit P38-MAPK (López-Carballo et al., 2002; Masiá et al., 2007; Pein et al., 2017). Furthermore, glutamate-induced cell death is associated with decreased levels of anti-apoptotic protein Bcl-2 (B-cell lymphoma 2) and increased levels of pro-apoptotic proteins including p53 (tumour protein P53), Bid (BH3 interacting-domain death agonist), and Bax (Bcl-2-like protein 4) (Dar et al., 2017). RA counteracts these effects through RARα, downregulating Bax and upregulating Bcl-2 expression levels under conditions of oxygen/glucose deprivation in PC12 cells (Zhang et al., 2014). These mechanisms collectively contribute to ellorarxine's protective action against glutamate-induced neuronal damage.

Ellorarxine may also have the potential to mitigate glutamate-induced excitotoxicity by influencing caspase-mediated cell death pathways, pivotal in apoptosis regulation across diverse cell types and conditions. In these pathways, nucleocytoplasmic translocation of mTOR triggers p53 phosphorylation, which in turn enhances the expression of pro-apoptotic proteins such as Bax, leading to cytochrome c release from mitochondria and subsequent activation of the caspase cascade culminating in neuronal death (Perfettini et al., 2005). RA has been reported to inhibit superoxide-induced activation of p38-MAPK and caspase-3, thereby fostering cell survival (Jameel et al., 2009).

The second system explored as a cause of neuronal death in neurodegenerative disease was dysregulation of proteostasis (protein homeostasis), which results in protein aggregation and dysfunctional lysosomal activity. Both processes play key roles in ALS pathogenesis (Robberecht and Philips, 2013). RA signalling pathways have been proposed to regulate protein homeostasis in cell culture and animal models by regulating the ubiquitin–proteasome system (UPS) and autophagy-lysosomal pathways (Chang et al., 2013; Riancho et al., 2016; Zhu et al., 2020). In this study, the influence of atRA and ellorarxine was determined under the stress condition of epoxomicin inhibition of the proteasome. Proteasome inhibition results in several changes that parallel ALS, including cytoplasmic protein aggregation, mitochondrial dysfunction, antioxidant exhaustion, and apoptosis (Boukhtouche et al., 2006; Cheng et al., 2013).

atRA has previously been shown to have neuroprotective effects following epoxomicin induced-proteasome inhibition in SH-SY5Y cells through stimulation of the PI3/Akt pathway (Cheng et al., 2013). In the NSC-34 motor neuron-like cell line used in this study, neuroprotection by ellorarxine against epoxomicin-induced toxicity appeared to depend on ERK1/2 pathway and partially on the PI3/Akt pathway, with the proviso that this may result from off-target effects of the Akt inhibitor used to act on ERK. Interestingly, atRA neuroprotection against epoxomicin-induced neurotoxicity appeared to be only based on the PI3/Akt pathway.

A third factor potentially influenced by the RAR regulatory system in the context of ALS is the response of mRNA metabolism to stress (Wolozin and Ivanov, 2019; Brown et al., 2020), particularly as manifested by changes in SGs. Stress granules form in response to heat shock or oxidative stress, such as that caused experimentally by sodium arsenite. After the stress source is removed, SGs typically disassemble quickly, serving as temporary and dynamic cytoplasmic storage sites for mRNA. However, dysregulation of this dynamic process can result in persistent and non-dynamic pathological SGs that promote aggregation and potentially lead to neurodegeneration (Wolozin and Ivanov, 2019; Brown et al., 2020).

To date, there has been limited research on the effect of the RA signalling pathway on SG formation and dynamics. Stress results in SGs containing RBPs and mRNA with weak and transient intermolecular interactions. Prolonged stress leads to the recruitment of non-translating messenger ribonucleoproteins (mRNPs) and RBPs to SG foci, forming a more stable and less dynamic SG core with a more dynamic and less stable peripheral shell. These physiological SGs remain in dynamic equilibrium with surrounding polysomes, and removing the stress source typically resolves the pathology and translation resumes. In contrast, dense and less dynamic pathological SGs, which are not in equilibrium with their surrounding environment, generate a non-fluid (gel-like) state. These pathological SGs, which are no longer transient (Advani and Ivanov, 2020), serve as a nidus for protein aggregation and sequester the RBPs, depleting nuclear RBPs and disrupting mRNA post-transcriptional modification and translation.

Pre-treatment of the NSC-34 cells with ellorarxine reduced both the number and size of the SGs compared to the DMSO (control) group. This suggests that ellorarxine delays SG initiation and formation and/or accelerates SG disassembly. Stress granule assembly is initially triggered by inhibition of translation initiation, which depends on pathways including the eIF4E-binding protein (4E-BPs)/mTOR pathway. Phosphorylation of 4E-BPs by mTOR inhibits their association with eIF4E, allowing translation to proceed. Concurrently, mTOR phosphorylates S6 kinase 1 and (S6K1) and S6 kinase 2 (S6K2), which are essential for SG formation and maintenance under stress conditions. S6K1 regulates translation suppression under stress conditions, while S6K2 regulates SG maintenance. The effect of mTOR on translation is a balance between S6K1 activity and 4E-BPs/mTOR pathway. Prolonged stress leads to mTOR sequestration into solid SGs, inhibiting mTOR activity and promoting translational arrest (Sfakianos et al., 2018).

RA has been shown to negatively regulate the mTOR pathway through RARα (Hsu et al., 2019; Long et al., 2021). Thus, the effect of RAR ligands on SG formation and maintenance may involve the inhibition of S6K1 and S6K2 phosphorylation through the mTOR pathway. Further investigation into how RAR ligands control SG assembly, dynamics, and disassembly will be important for understanding their potential to reduce motor neuron loss in ALS.

**Figure 7:**
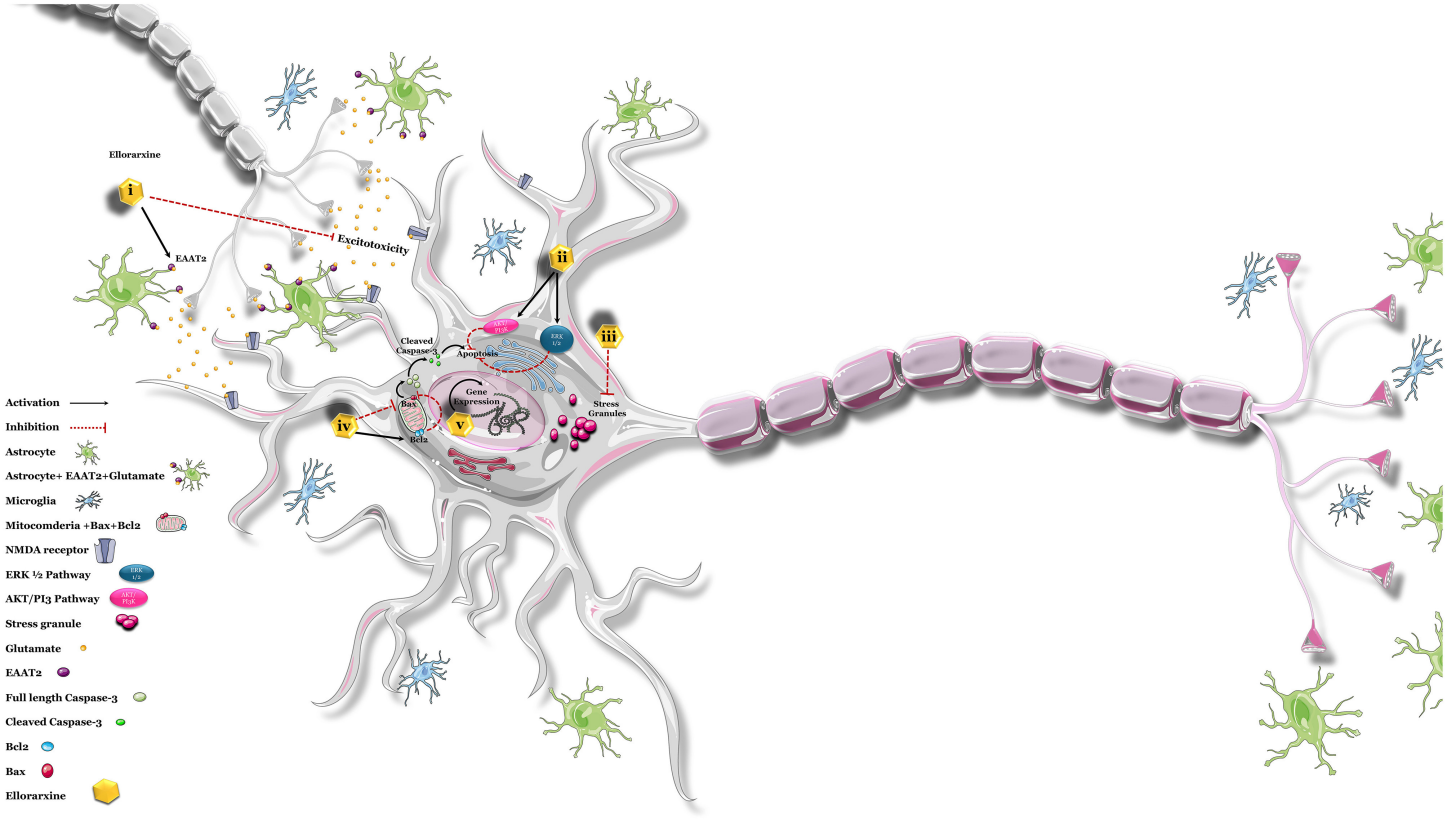
Possible neuroprotective effects of ellorarxine. The potential neuroprotective effects of ellorarxine may be mediated through several mechanisms: (i) regulating excitotoxicity: ellorarxine may increase the post-translational modification of EAAT2, enhancing its function and thereby reducing excitotoxicity. (ii) Restoring proteasome activity: by activating the PI3K/Akt and ERK1/2 pathways, ellorarxine may help restore proteasome activity, which is crucial for protein degradation and cellular homeostasis. (iii) Reducing stress granule (SG) formation: ellorarxine may negatively regulate mTOR, which in turn regulates protein translation, thus reducing the formation and maintenance of stress granules. (iv) Modulating apoptosis pathways: ellorarxine may decrease Bax levels and increase Bcl-2 levels, leading to the inhibition of apoptosis and the inactivation of the caspase-3 cascade. (v) Regulating gene expression: ellorarxine may influence the expression of genes associated with neuroprotection and neuroinflammation, such as Igf1 and Il1β.

The *in vitro* studies point to the neuroprotective action of ellorarxine in pathways linked with ALS. When injected into mice, the drug also concentrates in tissues such as the spinal cord, where it could support the survival of lower motor neurons. Ligands for the RARs have been proposed as a treatment for ALS and tested in animal models. For instance, nanoparticles containing RARβ agonist as a novel clinical approach for ALS treatment (Medina et al., 2020) increase lifespan and reduce neurodegeneration in SOD1G93A mice. Similarly, atRA (3 mg/kg) daily for 5 weeks from the onset stage of symptoms in SOD1G93A mice improved forelimb grip strength and slowed disease progression. It reduced the aggregation of misfolded proteins through the ubiquitin–proteasome system (UPS) system, blocked the activation of astrocytes, ameliorated neurological deficits, increased neurotrophic factor levels in the anterior horn of the spinal cord, and suppressed oxidative stress. Therefore, based on the above findings, induction of retinoic acid pathways may be proposed as a potential therapeutic target for ALS (Figure 7).

The robustness of ellorarxine's cytoprotective effect in our study is underscored by the consistent attenuation of neuronal cell death and modulation of stress granule formation across multiple stress conditions. Experimental conditions were primarily chosen to reflect key aspects of ALS pathogenesis, including excitotoxicity, proteostasis disruption, and oxidative stress-induced stress granule formation, although it is recognised that these cellular events are also disrupted in other neurodegenerative disorders. Our findings demonstrate a reproducible reduction in cellular damage and stress granule accumulation upon ellorarxine treatment compared to controls.

The limitations of atRA, such as its chemical instability and the inconsistent effects due to degradation into various isomers, underscore the importance of exploring more stable synthetic retinoids for therapeutic purposes. Ellorarxine among other novel RAR ligands we screened, offers improved stability and potentially enhanced efficacy without the variability seen with atRA. By using a stable compound like ellorarxine, we can better assess its neuroprotective capabilities and therapeutic potential in a consistent and controlled manner, paving the way for more effective treatments for ALS and potentially other neurodegenerative diseases.

## Data availability statement

The raw data supporting the conclusions of this article will be made available by the authors, without undue reservation.

## Ethics statement

The animal study was approved by University of Aberdeen Ethical Review Committee. The study was conducted in accordance with the local legislation and institutional requirements.

## Author contributions

AK: Writing – review & editing, Writing – original draft, Visualization, Validation, Resources, Methodology, Investigation, Formal analysis, Conceptualization. AW: Writing – review & editing, Writing – original draft. PM: Writing – review & editing, Writing – original draft, Supervision, Resources, Project administration, Funding acquisition, Conceptualization.
